# Distinct mutation profile and prognostic relevance in patients with hypoplastic myelodysplastic syndromes (h-MDS)

**DOI:** 10.18632/oncotarget.11050

**Published:** 2016-08-04

**Authors:** Chi-Yuan Yao, Hsin-An Hou, Tzung-Yi Lin, Chien-Chin Lin, Wen-Chien Chou, Mei-Hsuan Tseng, Ying-Chieh Chiang, Ming-Chih Liu, Chia-Wen Liu, Yuan-Yeh Kuo, Shang-Ju Wu, Xiu-Wen Liao, Chien-Ting Lin, Bor-Shen Ko, Chien-Yuan Chen, Szu-Chun Hsu, Chi-Cheng Li, Shang-Yi Huang, Ming Yao, Jih-Luh Tang, Woei Tsay, Chieh-Yu Liu, Hwei-Fang Tien

**Affiliations:** ^1^ Division of Hematology, Department of Internal Medicine, National Taiwan University Hospital, Taipei, Taiwan; ^2^ Department of Laboratory Medicine, National Taiwan University Hospital, Taipei, Taiwan; ^3^ Department of Pathology, National Taiwan University Hospital, Taipei, Taiwan; ^4^ Graduate Institute of Oncology, College of Medicine, National Taiwan University, Taipei, Taiwan; ^5^ Tai-Cheng Stem Cell Therapy Center, National Taiwan University, Taipei, Taiwan; ^6^ Biostatistics Consulting Laboratory, Department of Nursing, National Taipei College of Nursing, Taipei, Taiwan

**Keywords:** myelodysplastic syndromes, bone marrow hypocellularity, revised international prognostic scoring system, gene mutation, prognosis

## Abstract

Myelodysplastic syndromes (MDS) are a heterogeneous group of hematologic malignancies. Although most MDS patients have normal or increased BM cellularity (NH-MDS), some have hypocellular BM (h-MDS). The reports concerning the differences in genetic alterations between h-MDS and NH-MDS patients are limited. In this study, 369 MDS patients diagnosed according to the WHO 2008 criteria were recruited. h-MDS patients had lower PB white blood cell and blast counts, and lower BM blast percentages, than those with NH-MDS. h-MDS was closely associated with lower-risk MDS, defined by the International Prognostic Scoring System (IPSS) and revised IPSS (IPSS-R). IPSS-R could properly predict the prognosis in h-MDS (*P*<0.001) as in NH-MDS patients. The h-MDS patients had lower incidences of *RUNX1, ASXL1, DNMT3A, EZH2* and *TP53* mutations than NH-MDS patients. The cumulated incidence of acute leukemic transformation at 5 years was 19.3% for h-MDS and 40.4% for NH-MDS patients (*P=* 0.001). Further, the patients with h-MDS had longer overall survival (OS) than those with NH-MDS (*P=* 0.001), and BM hypocellularity remains an independent favorable prognostic factor for OS irrespective of age, IPSS-R, and gene mutations. Our findings provide evidence that h-MDS indeed represent a distinct clinico-biological subgroup of MDS and can predict better leukemia-free survival and OS.

## INTRODUCTION

Myelodysplastic syndromes (MDS), a heterogeneous group of clonal hematopoietic malignancies, are the most frequently encountered acquired bone marrow (BM) failure syndromes in adults [1, 2]. While the disease is classified as normo/hypercellular MDS (NH-MDS) in the majority of patients, about 10-20% of patients have hypoplastic MDS (h-MDS), characterized by a hypocellular BM, traditionally defined as less than 30% cellularity in the BM trephine biopsy specimens [3–5]. Recently, age-adjusted criteria of marrow hypocellularity have also been proposed to define h-MDS, for instance, <30% cellularity in patients younger than 70 years and <20% cellularity in patients older than 70 years, to account for the physiologically deceasing marrow cellularity with increasing age [6–8]. Although h-MDS share a number of similar clinical features with NH-MDS, and both have a propensity to leukemic transformation during clinical follow-up, h-MDS appear to be a distinct clinico-pathological entity [9, 10], suggesting an unique underlying pathogenesis of this disease. Recently, a number of somatic mutations, such as mutations in *ASXL1, EZH2, TET2, IDH1/2, DMNT3A, RUNX1, NRAS, KRAS, TP53*, and splicing complex genes, which may play important roles in the pathogenesis of MDS, have been described [11]. However, the reports concerning the differences in the genetic alterations between h-MDS and NH-MDS are limited. Further, although the revised international prognostic scoring system (IPSS-R) has been demonstrated to be more powerful than IPSS in prognostication for MDS patients [12, 13], its applicability to h-MDS remains to be explored. The aims of this study were to elucidate the differences in the clinical features, genetic abnormalities, and clinical outcomes between h-MDS and NH-MDS in a large cohort of MDS patients in Taiwan, and to verify the applicability of IPSS-R in this special entity of MDS.

## RESULTS

### Clinical and laboratory characteristics

A total of 369 MDS patients, diagnosed according to the 2008 WHO classification, were included in our study. Most patients (63.7%) in our cohort received mainly transfusions and/or other supportive care, while 20 (5.4%) patients received hypomethylating agents, 37 (10.0%) received low-dose chemotherapy, 26 (7.1%) received AML-directed intensive chemotherapy, and 51 (13.8%) underwent allogeneic hematopoietic stem cell transplantation. Among the total cohort, 100 (27.1%) patients were diagnosed as having h-MDS. The comparisons of clinical and laboratory features between patients with h-MDS and NH-MDS are listed in Table 1. Patients with h-MDS had lower peripheral blood (PB) white blood cell (WBC) counts (*P=* 0.030) and blast counts (*P*=0.006), and BM blast percentages (*P*=0.001) than those with NH-MDS. There was no difference in the age and gender distribution, hemoglobin, platelet, and serum lactate dehydrogenase levels between these two groups. h-MDS patients had statistically higher proportion of refractory cytopenia with unilineage dysplasia (RCUD) and refractory cytopenia with multilineage dysplasia (RCMD), but lower proportion of refractory anemia with excess blasts-1 (RAEB-1), compared with NH-MDS patients (28.0% *vs.* 17.5%, *P=* 0.029; 38.0% *vs.* 20.8%, *P=* 0.001; 11.0% *vs.* 24.9%, *P=* 0.004, respectively). h-MDS patients were classified more frequently to IPSS low and intermediate-1 risk groups (80.0% *vs.* 58.6%, *P*<0.001), or IPSS-R very low, low, and intermediate risk groups (72.2% *vs.* 52.6%, *P=* 0.001) than NH-MDS patients.

**Table 1:** Comparison of clinical and laboratory features between h-MDS and NH-MDS patients

Clinical characters	Total (n=369)	h-MDS (n=100, 27.1%)	NH-MDS (n=269, 72.9%)	*P* value
**Sex**				0.324
**Male**	242 (65.6%)	70 (70.0%)	172 (63.9%)	
**Female**	127 (34.4%)	30 (30.0%)	97 (36.1%)	
Age (year)*	65.2 (16.4-94.5)	61.6 (18.4-94.5)	65.3 (16.4-90.5)	0.997
Lab data**				
**WBC (/μL)**	3490 (490-40500)	3050 (650-9890)	3660 (490-40500)	0.030*
**Hb (g/dL)**	8.1 (3.4-14.6)	8.1 (3.7-14.4)	8.2 (3.2-14.6)	0.971
**Platelet (×1,000 /μL)**	77 (3-931)	66 (3-618)	82 (3-931)	0.179
**LDH (U/L)**	482 (145-6807)	463 (145-3122)	495 (210-6807)	0.361
**PB blast count (/μL)**	0 (0-3270)	0 (0-523)	0 (0-3270)	0.006*
**BM blast %**	3.2 (0-19.0)	2.0 (0-19.0)	4.5 (0-19.5)	0.001*
**2008 WHO classification**^#^				
**RCUD**	75 (20.3%)	28 (28.0%)	47 (17.5%)	0.029*
**RARS**	20 (5.4%)	3 (3.0%)	17 (6.3%)	0.302
**RCMD**	94 (25.5%)	38 (38.0%)	56 (20.8%)	0.001*
**RCMD-RS**	14 (3.8%)	3 (3.0%)	11 (4.1%)	0.767
**RAEB1**	78 (21.1%)	11 (11.0%)	67 (24.9%)	0.004*
**RAEB2**	85 (23.1%)	17 (17.0%)	68 (25.3%)	0.097
**MDS-U**	3 (0.8%)	0 (0%)	3 (1.1%)	0.566
**IPSS**^‡^^#^^§^				<0.001*
**Low+INT-1**	219 (64.2%)	72 (80.0%)	147 (58.6%)	
**INT-2+High**	122 (35.8%)	18 (20.0%)	104 (41.4%)	
**IPSS-R**^‡^^#^^ζ^				0.001*
**Very low+low+INT**	197 (57.8%)	65 (72.2%)	132 (52.6%)	
**High+very high**	144 (42.2%)	25 (27.8%)	119 (47.4%)	
**Treatment modalities**				
**Transfusion/BCS**	235 (63.7%)	70 (70.0%)	165 (61.3%)	0.144
**HMA**	20 (5.4%)	4 (4.0%)	16 (5.9%)	0.608
**Intensive chemotherapy**	26 (7.1%)	2 (2.0%)	24 (8.9%)	0.021*
**Lose-dose chemotherapy**	37 (10.0%)	7 (7.0%)	30 (11.2%)	0.329
**HSCT**	51 (13.8%)	17 (17.0%)	34 (12.6%)	0.309

** Median (range).

* Statistically significant if *P*<0.05.

# Number of patients (% of patients within either hypoplastic or non-hypoplastic MDS subgroups).

‡ 341patients, including 90 h-MDS and 251 NH-MDS patients, had chromosome data at diagnosis.

§ IPSS: Low, 0; intermediate (INT)-1, 0.5-1; INT-2, 1.5-2; and High, ≥ 2.5.

ζ IPSS-R: Very low, ≦1.5; Low, >1.5-3; intermediate (INT),>3-4.5; High, >4.5-6; and Vey high, >6.

Abbreviations: h-MDS, hypoplastic MDS; NH-MDS, normo-/hypercellular MDS; FAB, French-American-British classification; RARS, refractory anemia with ring sideroblasts; RAEB, refractory anemia with excess blasts; RCUD, refractory cytopenia with unilineage dysplasia; RCMD, refractory cytopenia with multilineage dysplasia; and MDS-U, MDS (unclassifiable); IPSS, international prognosis scoring system; IPSS-R, revised IPSS.

### Comparison of cytogenetic changes and genetic alterations between h-MDS and NH-MDS

Clonal chromosomal abnormalities were detected in 154 (45.2%) of 341 patients who had chromosomal data at diagnosis (Supplementary Table S2). The incidence of cytogenetic abnormalities was similar between the h-MDS and NH-MDS groups (42.2% *vs.* 46.2%, *P=* 0.815). We found that h-MDS patients had statistically lower incidence of poor-risk cytogenetic abnormalities than the NH-MDS patients (7.8% vs. 24.3%, *P*<0.001). However, there was no difference in the distribution of any specific chromosomal abnormalities between patients with h-MDS and NH-MDS.

To investigate the interaction of genetic alterations in the pathogenesis of h-MDS, a mutational screening of 17 genes was performed (Table 2). Overall, 203 (55.0%) patients had at least one gene mutation, 35 (35.0%) in the h-MDS group, compared to 168 (62.5%) in NH-MDS group (*P*<0.001). The most common mutation in h-MDS was *SF3B1* mutation (12.0%), followed by *TET2* (11.4%), and *ASXL1* mutations (7.1%). Patients with h-MDS had significantly lower incidences of *RUNX1, ASXL1, DNMT3A, EZH2*, and *TP53* mutations than those with NH-MDS (4.0% *vs.* 14.2%, *P=* 0.005; 7.1% *vs.* 21.7%, *P*=0.001; 3.0% *vs.* 12.6%, *P=* 0.006; 0% *vs.* 5.2%, *P=* 0.014, 3.0% *vs.* 10.8%, *P=* 0.020, respectively). If we consider cytogenetic abnormalities and molecular alterations collectively, 262 (71.0%) of the total cohort had at least one genetic aberration, 57 (57.0%) in the h-MDS subgroup and 205 (76.2%) in the NH-MDS subgroup (*P*<0.001).

**Table 2:** Comparison of genetic alterations between patients with h-MDS and NH-MDS

Variables	Number examined	Total cohort (%)	h-MDS (%)	NH-MDS (%)	*P* value
		**Mutated**	**Mutated**	**Mutated**	
***FLT3/* ITD**	366	1.1%	1.0%	1.1%	>0.999
***NRAS***	369	2.2%	1.0%	2.6%	0.688
***KRAS***	367	1.1%	0%	1.5%	0.578
***JAK2***	368	0.8%	1.0%	0.7%	>0.999
***RUNX1***	367	11.4%	4.0%	14.2%	0.005*
***MLL/* PTD**	352	0.6%	0%	0.8%	>0.999
***IDH1***	368	0.5%	1.0%	0.4%	0.470
***IDH2***	366	2.2%	0%	3.0%	0.113
***ASXL1***	366	17.8%	7.1%	21.7%	0.001*
***TET2***	282	12.4%	11.4%	12.7%	>0.999
***DNMT3A***	369	10.0%	3.0%	12.6%	0.006*
***TP53***	369	8.7%	3.0%	10.8%	0.020*
***SETBP1***	369	2.4%	1.0%	3.0%	0.454
***EZH2***	369	3.8%	0%	5.2%	0.014*
***SF3B1***	369	11.4%	12.0%	11.2%	0.854
***U2AF1***	369	7.9%	5.0%	8.9%	0.278
***SRSF2***	369	10.8%	6.0%	12.6%	0.089

* Statistically significant if *P*<0.05.

### Survival analysis

**Figure 1:**
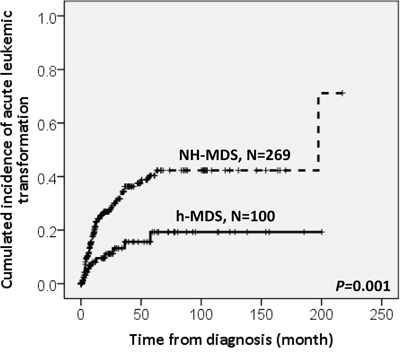
The cumulated incidence of acute leukemic transformation at 5 years was significantly lower in h-MDS patients (19.3%) than in NH-MDS patients (40.4%)

**Figure 2:**
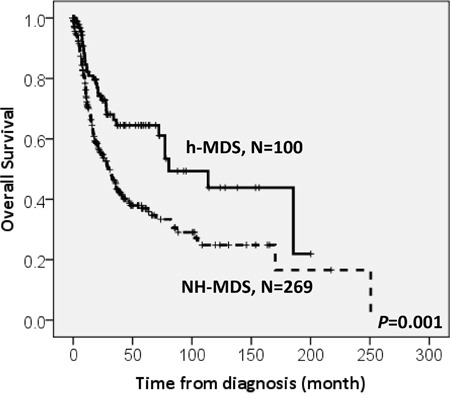
The comparison of overall survival between patients with h-MDS and NH-MDS Patients with h-MDS had a longer median overall survival than those with NH-MDS (80.5 months *vs.* 29.6 months, *P*=0.001).

**Figure 3:**
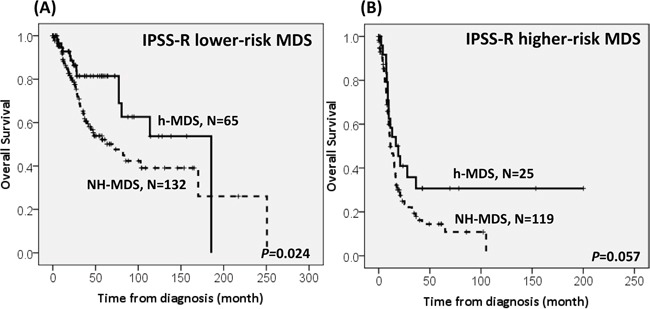
The comparison of overall survival between h-MDS and NH-MDS patients in subgroups of patients with lower-risk and higher-risk MDS The survival difference between patients with h-MDS and NH-MDS remains statistically significant in lower-risk MDS (IPSS-R very low, low and intermediate risks), with a median OS of 185.5 months and 69.9 months, respectively **A.**, but not among higher-risk MDS (IPSS-R high and very high risks), with a median OS of 16.8 months and 11.4 months, respectively **B.**

**Figure 4:**
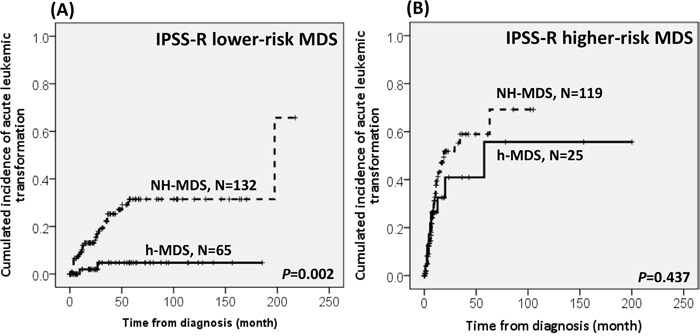
The comparison of the risk of acute leukemic transformation between h-MDS and NH-MDS patients in subgroups of patients with lower-risk and higher-risk MDS The difference in the incidence of acute leukemic transformation between patients with h-MDS and NH-MDS remains statistically significant in lower-risk MDS, with a 5-year acute leukemic transformation rate of 4.8% and 31.4%, respectively **A.**, but not among higher-risk MDS, with an acute leukemic transformation rate of 55.7% and 59.0%, respectively **B.**

**Figure 5:**
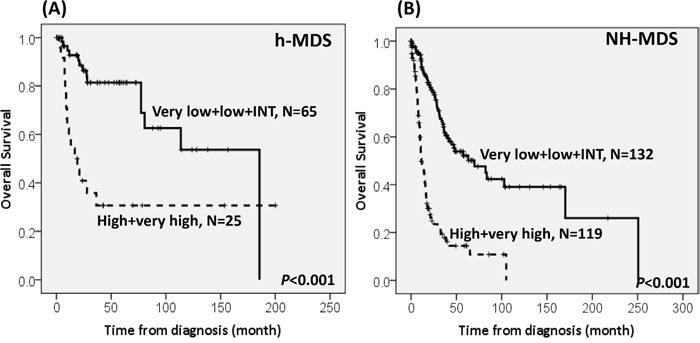
The Kaplan-Meier survival curves of h-MDS and NH-MDS patients, stratified by IPSS-R The IPSS-R can well predict the survival in h-MDS patients, with the median overall survival of 185.5 months *vs*. 16.8 months in the IPSS-R lower-risk (very low, low and intermediate risks) and higher-risk (high and very high risks) subgroups, respectively **A.**, the same as in NH-MDS patients, with the median OS of 69.9 months *vs*. 11.4 months in the IPSS-R lower-risk and higher-risk subgroups, respectively **B.**

With a median follow-up duration of 46.9 months (range, 0.1-250.7 months), the cumulated incidence of acute leukemic transformation at 5 years was 19.3% for h-MDS patients and 40.4% for NH-MDS patients (*P=* 0.001, Figure 1). In addition, the patients with h-MDS had a longer overall survival (OS) than those with NH-MDS (median, 80.5 months *vs.* 29.6 months, *P*=0.001, Figure 2). In subgroup analysis, the survival difference between h-MDS and NH-MDS patients remained statistically significant only among the patients with lower-risk MDS (IPSS-R very low, low and intermediate risks, median 185.5 months *vs*. 69.9 months, *P=* 0.024, Figure 3A), but not in those with higher-risk MDS (IPSS-R high and very high risks, median 16.8 months *vs*. 11.4 months, *P*=0.057, Figure 3B). Similarly, the lower incidence of acute leukemic transformation in h-MDS, compared to NH-MDS, remained significant only in the lower-risk group (4.8% *vs.* 31.4%, *P=* 0.002, Figure 4A), but not in the higher-risk group (55.7% *vs.* 59.0%, *P*=0.437, Figure 4B). The IPSS-R could well predict the survival of h-MDS patients: patients with lower-risk MDS had a significantly longer median OS than those with higher-risk MDS (185.5 months *vs.* 16.8 months, *P*<0.001, Figure 5A), the same as in NH-MDS patients (69.9 months *vs.* 11.4 months, *P*<0.001, Figure 5B). In multivariate analysis, we included gene mutation variables with *P* values less than 0.1 in the univariate analysis, plus relevant clinical parameters, such as age, sex, BM cellularity, IPSS-R risk stratification, and treatment modality. We found that the BM hypocellularity was an independent favorable prognostic factor for OS (relative risk 0.655, 95% CI 0.431-0.995, *P*=0.047) irrespective of age, sex, IPSS-R, gene mutations, and treatment modality. (Table 3).

**Table 3:** Multivariate analysis (Cox regression) for the overall survival in 369 MDS patients

Variable	RR	Overall survival		*P* value
		Lower 95% CI	Upper 95% CI	
**Age†**	1.533	1.035	2.273	0.033*
**Sex**	1.353	0.957	1.912	0.087
**BM Hypocellularity**	0.655	0.431	0.995	0.047*
**IPSS-Rζ**	3.431	2.404	4.896	<0.001*
***TP53***	5.904	3.442	10.130	<0.001*
***ASXL1***	1.394	0.892	2.179	0.144
***EZH2***	1.066	0.479	2.371	0.876
***DNMT3A***	1.367	0.847	2.204	0.200
***RUNX1***	0.983	0.608	1.590	0.946
***SRSF2***	1.440	0.838	2.474	0.187
**HSCT or intensive chemotherapy§**	0.994	0.643	1.535	0.977

* Statistically significant if *P*<0.05.

† Age > 65 relative to Age ≤ 65 (the reference).

ζ Higher-risk *vs.* Lower-risk.

§ HSCT or intensive chemotherapy *vs.* others.

Abbreviations: RR, Relative Risk; CI, confidence interval.

## DISCUSSION

We found that h-MDS were associated with distinct clinico-biological features and mutation profile, and that BM hypocellularity represented a favorable prognostic factor in the MDS patients, independent of age, sex, IPSS-R and genetic alterations. Further, IPSS-R could hold its predictive power of discriminating prognosis in the h-MDS patients, as in the NH-MDS patients.

In this study, 27.1% MDS patients had h-MDS, a percentage higher than those (10-20%) reported in literature [3, 4, 6, 14–16]. We speculate that the difference could be explained by the following reasons. First, in some of the previous reports, chronic myelomonocytic leukemia (CMMoL) and refractory anemia with excess blasts in transformation (RAEB-T) were not excluded [4, 7, 8]. Therefore, the higher percentage of the h-MDS patients in this study might represent a renewed epidemiology in the contemporary era of the WHO classification. Second, in our study, we included only the “*de novo*” MDS patients; while in other studies, therapy-related MDS patients were either enrolled altogether, or not specifically excluded [6, 7, 17]. Third, differences in the genetic and environmental backgrounds and clinico-pathological features of the MDS patients between Asian and Western countries have been reported [13, 18–20], which might contribute to the difference in h-MDS prevalence as well.

To date, there has been no universal consensus on whether h-MDS belong to the lower- or higher-risk subset of MDS. Yue et al reported there was no difference in the distribution of IPSS risk scores between the h-MDS and NH-MDS patients [6], while others demonstrated that the h-MDS patients more frequently had IPSS lower-risk MDS [8], or on the contrary, IPSS higher-risk MDS [7]. In this large homogeneous cohort of consecutive unselected *de novo* MDS patients classified by the 2008 WHO criteria, we distinctly demonstrated that h-MDS was associated with lower-risk subgroups in both IPSS and IPSS-R, a finding not unequivocally shown previously. The utility of IPSS in the risk stratification of patients with h-MDS has been reported before, both in Asian and Western populations [6, 9]. However, Tong et al. [7], proposed a new prognostic scheme for h-MDS patients because they found IPSS failed to distinguish the outcome of these patients [7]. In 2012, IPSS-R was introduced to better risk-stratify *de novo* MDS patients [12]. To our knowledge, no study has specifically examined the power of IPSS-R in risk stratification of h-MDS patients. To shed light on the applicability of IPSS-R in this setting, we analyzed the survival of our h-MDS patients according to the IPSS-R, and found that IPSS-R did successfully stratify h-MDS patients into distinct risk groups (Figure 5A).

Although AA and h-MDS share the same hypocellularity in the BM biopsy specimen, the combination of dysplasia of hematopoietic cells, and clonal cytogenetic abnormality can allow a conclusive diagnosis of MDS. In our cohort, the morphologic diagnosis of MDS was made according to the 2008 WHO classification, and all the h-MDS cases had BM morphologies fulfilling the criteria of dysplasia. Besides, the incidence of clonal chromosomal abnormalities was similar between patients with h-MDS and NH-MDS. Taken above, the possibility that we inadvertently included AA patients into our h-MDS cohort should be negligible. On a more detailed inspection, although 5q deletion (as the sole abnormality) appeared more frequently, and loss of chromosome 7 (as the sole abnormality) less frequently in the h-MDS subgroup, the differences did not reach statistical significance (*P=* 0.073 and *P=* 0.199, respectively). There were no significant differences in other chromosomal abnormalities between the h-MDS and NH-MDS patients, either, a finding similar to what previous studies had reported [3, 4, 6, 21]. Gene mutations have started to be taken into account for risk stratification of myeloid malignancies recently, and cumulating data are suggesting that certain gene mutations have prognostic significance in MDS [22, 23]. Of particular interest, Nazha et al. recently reported the genomic analysis of 237 MDS patients, in which h-MDS patients comprised 14% [10]. In their study, there was no difference in the distribution of cytogenetic abnormalities between their NH-MDS and h-MDS subgroups. The patients with h-MDS had a lower average number of somatic mutations, and lower incidences of *SF3B1* and *IDH1/2* mutations. However, they did not find a survival difference between patients with h-MDS and NH-MDS. In our current study, we examined the mutational status of 17 genes relevant to myeloid malignancies, and found that patients with h-MDS had lower number of concurrent genetic alterations, similar to the report by Nazha et al. However, in contrast to their report, we could not find a negative association of *SF3B1* and *IDH1/2* mutations with h-MDS. Instead, we showed that the h-MDS subgroup had less frequent *RUNX1, ASXL1, DNMT3A, EZH2*, and *TP53* mutations compared with the NH-MDS subgroup. These findings may be of prognostic significance, since mutations in the aforementioned genes have been linked to the pathogenesis, disease progression, and inferior survival of MDS patients [24–37]. In other words, although BM hypocellularity was an independent prognostic factor, the lower frequencies of the poor-risk gene mutations in the h-MDS patients might also contribute to the better survival of this group of patients.

Whether the prognosis of the h-MDS patients differs from that of the NH-MDS patients remains unsettled. Our group previously reported that h-MDS predicted a favorable outcome in a cohort of 189 patients defined by the FAB classification [9], similar to the report by Yue et al [6], while other researchers did not observe such survival benefit [3, 4, 7, 8, 10, 17]. The reason of the variability in prognosis is unknown, but may be due to differences in the ethnic backgrounds, specific patient populations selected (age ranges and FAB subtypes, etc.) and treatment regimens. In this study, we showed that patients with h-MDS had a lower incidence of leukemia transformation and longer OS than those with NH-MDS. And, intriguingly, the prognostic relevance remained significant only in the lower-risk, but not in the higher-risk MDS patients. We postulate that this finding is of particular importance clinically, because although the IPSS and IPSS-R remain two of the most commonly used prognostic scoring systems for MDS [38], the median survival and risk of AML progression may still vary within the same risk group, especially the lower-risk ones [39]. There is clearly an unmet need to identify patients with IPSS/IPSS-R lower-risk MDS that may have an increased risk for disease progression and shortened survival. The fact that we distinctly showed BM hypocellularity is an independent favorable prognostic factor in the MDS patients and that BM cellularity alone could further stratify the lower-risk MDS patient into discrete prognostic subgroups, suggests that BM cellularity could potentially fulfill the unmet need in this group of patients.

In summary, we provide evidence that h-MDS are a distinct clinico-biological subgroup of MDS. h-MDS occur more frequently in patients with lower-risk MDS, have lower number of concurrent gene mutations, and are inversely associated with *RUNX1, ASXL1, DNMT3A, EZH2*, and *TP53* mutations. We demonstrate that BM hypocellularity is an independent favorable prognostic factor which could further risk-stratify MDS patients, especially the lower-risk group. Further prospective studies in larger cohorts of MDS patients are warranted to validate the unique properties and prognostic relevance of h-MDS.

## MATERIALS AND METHODS

### Patients

A total of 369 adult patients diagnosed consecutively with *de novo* MDS according to the 2008 WHO classification [40] between 1990 and 2010 at the National Taiwan University Hospital were recruited. The clinical features, laboratory data, and BM findings were reviewed. This study was approved by the Institutional Review Board of the National Taiwan University Hospital; and written informed consents were obtained from all participants in accordance with the Declaration of Helsinki.

### Bone marrow aspiration and biopsy

BM aspirate smears were routinely stained with modified Wright–Giemsa stain (Liu's stain) at our institution [41], and biopsy specimens were stained with hematoxylin-eosin after decalcification. BM hypoplasia was assessed based on BM biopsies, and defined as<30% cellularity in patients younger than 70 years and <20% cellularity in patients older than 70 years. The morphological criteria for BM dyspoiesis included megaloblastoid changes, irregularly shaped nuclei or karyorrhexis of erythroblasts, micromegakaryocytes, hypolobated or binucleated megakaryocytes, and pseudo Pelger-Huët anomaly or hypogranulation of granulocytes [40, 42]. Any of the dysplastic features should be present in at least 10% of that particular cell lineage in order to be considered significant.

### Cytogenetics

BM cells were harvested directly or after 1-3 days of un-stimulated culture by procedures that had been described previously [43]. Metaphase chromosomes were banded by trypsin-Giemsa technique and karyotyped according to the International System for Human Cytogenetic Nomenclature [44].

### Mutation analysis

Analyses of mutations in 17 relevant genes, including *FLT3/* ITD [45], *NRAS* [45], *KRAS* [45], *JAK2* [45], and *RUNX1* [46], those genes related to epigenetic regulation, such as *MLL/* PTD [47], *ASXL1* [48], *IDH1* [49], *IDH2* [50], *EZH2* [51], *TET2* [52] and *DNMT3A* [53], and genes related to the RNA splicing machinery, such as *SF3B1* [54], *U2AF1* [55], and *SRSF2* [56], as well as *TP53*[57] and *SETBP1* [58], were performed as previously described. Please refer to Supplementary Table S1 for a detailed illustration of the sequences and genomic coordinates in these 17 genes. Abnormal sequencing results were confirmed by at least two repeated analyses.

### Statistics

Mann-Whitney U test was employed to compare continuous variables such as age and hemogram between h-MDS and NH-MDS patients. Pearson's chi-square test was utilized to detect the difference of nominal variables such as gender, WHO Classification, cytogenetic changes, IPSS and IPSS-R distribution and genetic alterations between h-MDS and NH-MDS patients. Fisher's exact test was used as required. OS was measured from the date of initial diagnosis to the date of last follow-up or death from any cause. We used Cox proportional hazards model for multivariate regression analysis. Kaplan-Meier analysis with log-rank test was performed to evaluate the difference in acute leukemic transformation rate and OS between groups. *P* values less than 0.05 were considered statistically significant. All statistical analyses were performed with the SPSS 17 (SPSS Inc., Chicago, IL, USA) and Statsdirect (Cheshire, England, UK).

## SUPPLEMENTARY TABLES




