# Supplementary material for: Distinct mutation profile and prognostic relevance in patients with hypoplastic myelodysplastic syndromes (h-MDS)
Source: Oncotarget. 2016 Aug 4;7(39):63177–88. doi: 10.18632/oncotarget.11050 (PMC5325355; doi:10.18632/oncotarget.11050)
Supplement: Supplementary file 1 [file oncotarget-07-63177-s001.pdf]

Distinct mutation profile and prognostic relevance in patients with hypoplastic myelodysplastic syndromes (h-MDS)

SUPPLEMENTARY TABLE

Supplementary Table S1: The sequence and genomic coordinates for amplification and sequencing primers in these 17 genes

See Supplementay File 1

Supplementary Table S2: Comparison of cytogenetic changes between h-MDS and NH-MDS patients

Variables	Total[‡]	h-MDS[‡]	NH-MDS[‡]	*P* value
**Normal karyotype**	187 (54.8%)	52 (57.8%)	135 (53.8%)	0.815
**Any karyotype abnormality**	154 (45.2%)	38 (42.2%)	116 (46.2%)	
**Karyotype risk**[†,#]				
**Good**	205 (60.2%)	59 (65.5%)	146 (58.2%)	0.480
**Intermediate**	68 (19.9%)	24 (26.7%)	44 (17.5%)	0.098
**Poor**	68 (19.9%)	7 (7.8%)	61 (24.3%)	**<0.001**
**Loss Y***	5 (1.5%)	2 (2.2%)	3 (1.2%)	0.615
**Del 20q***	10 (2.9%)	3 (3.3%)	7 (2.8%)	0.734
**Del 5q***	2 (0.6%)	2 (2.2%)	0 (0%)	0.073
**Trisomy 8***	17 (5.0%)	3 (3.3%)	14 (5.6%)	0.577
**Monosomy 7***	13 (3.8%)	1 (1.1%)	12 (4.8%)	0.199
**Other abnormalities**	107 (31.4%)	27 (30.0%)	80 (31.8%)	0.699

[‡]Cytogenetic data at diagnosis were available in 341 patients among total cohort, including 90 patients with h-MDS and 251 with NH-MDS.

[†]Good, normal karyotype, isolated -Y, del(5q) or del(20q); Poor, complex (≧ 3 abnormalities) or chromosome 7 anomalies; Intermediate, other abnormalities.

[#]Number of patients (% of patients within either hypoplastic or non-hypoplastic MDS subgroups)

*As the sole abnormality.
