# Supplementary material for: Distinct mutation profile and prognostic relevance in patients with hypoplastic myelodysplastic syndromes (h-MDS)
Source: Oncotarget. 2016 Aug 4;7(39):63177–88. doi: 10.18632/oncotarget.11050 (PMC5325355; doi:10.18632/oncotarget.11050)
Supplement: Supplementary file 2 [file oncotarget-07-63177-s002.docx]

**Supplementary Table 1.**

**The sequence and genomic coordinates for amplification and sequencing primers in these 17 genes**

| **Gene** | **Chromosome** | **Exons** | **Primer ID** | **Genomic start** | **Genomic stop** | **primer 1** | **primer 2** |
| --- | --- | --- | --- | --- | --- | --- | --- |
| *SF3B1* | 2 | 14 | 1 | 197,402,479 | 197,402,980 | AAGGCCGAGAGATCATTTCTAA | AGTCCAGTCTGGGCAACATAGT |
|  | 2 | 15-16 | 2 | 197,401,689 | 197,402,245 | ATGTGAACATATTCTGCAGTTTG | TGTTAGAACCATGAAACATATCCA |
| *U2AF1* | 21 | 2 | 1 | 43,104,148 | 43,104,547 | AAGCGGCCGCAGTCGATCACCTGCCTCACT | AAGCGGCCGCGGAGGTGCTTAATACCACGG |
|  | 21 | 6 | 2 | 43,094,630 | 43,094,838 | AAGTCTTATTAAAGCGTGGATGGC | ACGTCACTGGCCACTCCTCACTCAC |
| *SRSF2* | 17 | 2 |  | 76,736,774 | 76,736,974 | CACGACAAGCGCGACGCTGAGGA | CCGCGGTCCCCTCAGCCCCGTTTAC |
| *FLT3*/ITD | 13 | 14-15 |  | 28,033,883 | 28,034,214 | CAATTTAGGTATGAAAGCC | GTACCTTTCAGCATTTTGAC |
| *NRAS* | 1 | 2 | 1 | 114,716,044 | 114,716,227 | GTACTGTAGATGTGGCTCGC | CCTCACCTCTATGGTGGGAT |
|  | 1 | 3 | 2 | 114,713,733 | 114,714,000 | CCCTTACCCTCCACACCC | CCTCATTTCCCCATAAAGATTC |
| *KRAS* | 12 | 2 | 1 | 25,245,234 | 25,245,573 | GATACACGTCTGCAGTCAACTG | GGTCCTGCACCAGTAATATGC |
|  | 12 | 3 | 2 | 25,227,154 | 25,227,453 | GGTGCACTGTAATAATCCAGACT | CATGGCATTAGCAAAGACTCA |
| *PTPN11* | 12 | 3 | 1 | 112,450,288 | 112,450,575 | CTTGCCTCCCTTTCCAATGGAC | GCATTTCTGACACTCAGGGCAC |
|  | 12 | 13 | 2 | 112,488,879 | 112,489,234 | CAACACTGTAGCCATTGCAACA | CGTATCCAAGAGGCCTAGCAAG |
| *JAK2* | 9 |  | Normal | 5,073,580 | 5,073,943 | ATCTATAGTCATGCTGAAAGTAGGAGAAAG | CTGAATAGTCCTACAGTGTTTTCAGTTTCA |
|  | 9 |  | Mutated |  |  | AGCATTTGGTTTTAAATTATGGAGTATATT | CTGAATAGTCCTACAGTGTTTTCAGTTTCA |
| *WT1* | 11 | 7 | 1 | 32,396,119 | 32,396,468 | GACCTACGTGAATGTTCACATG | ACCAACACCTGGATCAGACCT |
|  | 11 | 9 | 2 | 32,391,836 | 32,392,184 | TGCAGACATTGCAGGCATGGCAGG | GCACTATTCCTTCTCTCAACTGAG |
| *RUNX1* | 21 | 3 | 1 | 34,886,813 | 34,887,150 | AGCTGTTTGCAGGGTCCTAA | GTCCTCCCACCACCCTCT |
|  | 21 | 4 | 2 | 34,880,515 | 34,880,848 | CATTGCTATTCCTCTGCAACC | CCATGAAACGTGTTTCAAGC |
|  | 21 | 5 | 3 | 34,859,396 | 34,859,621 | CCACCAACCTCATTCTGTTT | AGACATGGTCCCTGAGTATA |
|  | 21 | 6 | 4 | 34,834,296 | 34,834,798 | GGGGGCCCATTCTGCTGAGAGG | GAGCATCAAGGGGAAACCCC |
|  | 21 | 7 | 5 | 34,799,266 | 34,799,509 | AATCCCACCCCACTTTACAT | CTCAGCTGCAAAGAATGTGT |
|  | 21 | 8 | 6 | 34,792,055 | 34,792,639 | TCCGCTCCGTTCTCTTGC | GCTTGTCGCGAACAGGAG |
| *MLL*/PTD | 13 |  | ALOX5AP | 30,734,138 | 30,735,604 | TCTGGAGAGCCTTGGCTAATA | TTTGCTTCAGGCTCCCCA |
|  | 11 |  | MLL | 118,482,422 | 118,468,802 | GTCCAGAGCAGAGCAAACAG | CGCACTCTGACTTCTTCATC |
| *ASXL1* | 20 | 12 | 1 | 32,434,381 | 32,434,941 | AGGTCAGATCACCCAGTCAGTT | TAGCCCATCTGTGAGTCCAACTGT |
|  | 20 | 12 | 2 | 32,434,866 | 32,435,423 | AGAGGACCTGCCTTCTCTGAGAAA | TTCGATGGGATGGGTATCCAATGC |
|  | 20 | 12 | 3 | 32,435,363 | 32,435,894 | ACTTGAAAACCAAGGCTCTCGT | GCAACCATCCCATCTGTCCTTGTA |
|  | 20 | 12 | 4 | 32,435,796 | 32,436,469 | GGTGGACAAGGATGAGAAACCCAA | TGTCCTGTGACATAGCACGGACTT |
|  | 20 | 12 | 5 | 32,436,398 | 32,436,930 | TGGATTCCAAAGAGCAGTTCTCTTC | CATGACAAAGGGCATCCCTTCCAA |
|  | 20 | 12 | 6 | 32,436,863 | 32,437,455 | ACAGGAAAGCTACTGGGCATAGTC | CAAGAGTGCTCCTGCCTAAAGAGT |
| *IDH1* | 2 | 4 |  | 208,248,153 | 208,248,633 | TGTGTTGAGATGGACGCCTATTTG | TGCCACCAACGACCAAGTCA |
| *IDH2* | 15 | 4-5 |  | 90,088,245 | 90,088,803 | CTGCCTCTTTGTGGCCTAAG | ATTCTGGTTGAAAGATGGCG |
| *TET2* | 4 | 3 | 1 | 105,233,772 | 105,234,665 | TGCTATGTCTAGGTATTCCGA | CGCAATGGAAACACAATCTG |
|  | 4 | 3 | 2 | 105,234,288 | 105,235,123 | CAAAAGGCTAATGGAGAAAGACGTA | GCAGAAAAGGAATCCTTAGTGAACA |
|  | 4 | 3 | 3 | 105,234,852 | 105,235,694 | GCCAGTAAACTAGCTGCAATGCTAA | TGCCTCATTACGTTTTAGATGGG |
|  | 4 | 3 | 4 | 105,235,459 | 105,236,325 | GACCAATGTCAGAACACCTCAA | TTGATTTTGAATACTGATTTTCACCA |
|  | 4 | 3 | 5 | 105,236,097 | 105,236,884 | TTGCAACATAAGCCTCATAAACAG | ATTGGCCTGTGCATCTGACTAT |
|  | 4 | 3 | 6 | 105,236,695 | 105,237,458 | GGTACTTGATACATAACCATGC | TGCTGCCAGACTCAAGATTTAAA |
|  | 4 | 4 | 7 | 105,241,025 | 105,241,519 | TGTTTACTGCTTTGTGTGTGAAGG | ATACTACATATAATACATTCTAATTCCCTCACTG |
|  | 4 | 5 | 8 | 105,242,718 | 105,243,003 | CATTTCTCAGGATGTGGTCATAGAAT | CCCAATTCTCAGGGTCAGATTTA |
|  | 4 | 6 | 9 | 105,243,258 | 105,243,856 | AGACTTATGTATCTTTCATCTAGCTCTGG | ACTCTCTTCCTTTCAACCAAAGATT |
|  | 4 | 7 | 10 | 105,259,487 | 105,259,848 | ATGCCACAGCTTAATACAGAGTTAGAT | TGTCATATTGTTCACTTCATCTAAGCTAAT |
|  | 4 | 8 | 11 | 105,261,620 | 105,261,973 | GATGCTTTATTTAGTAATAAAGGCACCA | TTCAACAATTAAGAGGAAAAGTTAGAATAATATTT |
|  | 4 | 9 | 12 | 105,269,470 | 105,269,830 | AAATTACCCAGTCTTGCATATGTCTT | TGTCATTCCATTTTGTTTCTGGATA |
|  | 4 | 10 | 13 | 105,272,467 | 105,273,100 | CACAAATCTGAATACTGAGAGG | CTAGGTAATGGGTTGATCTG |
|  | 4 | 11 | 14 | 105,274,910 | 105,275,657 | GCTCTTATCTTTGCTTAATGGGTGT | TGTACATTTGGTCTAATGGTACAACTG |
|  | 4 | 11 | 15 | 105,275,410 | 105,276,250 | AATGGAAACCTATCAGTGGACAAC | AGCCATGTTTTGGCTCATTC |
|  | 4 | 11 | 16 | 105,276,064 | 105,276,617 | CAGAGCTTTCTGGATCCTGACAT | ACTGTGACCTTTCCCCACTG |
| *DNMT3A* | 2 | 2 | 1 | 25,313,814 | 25,314,249 | TCACAGGCCAGGTGTGGCCCTGGG | GCGGTCATGCACTCAGTATGAGG |
|  | 2 | 3 | 2 | 25,300,027 | 25,300,380 | ACAGCCCTGGAAGTGTGGCTCGTG | CTGGTCTTAAATGTCTCCAGGTC |
|  | 2 | 4 | 3 | 25,282,341 | 25,282,792 | ATTTCAGAGCGGTCAATGATCC | CCAGACCATCCTTCCTGGGACC |
|  | 2 | 5 | 4 | 25,275,421 | 25,275,630 | GACCCCAGGCACAAGGTG | GCCCCCTCACACACACTC |
|  | 2 | 6 | 5 | 25,274,854 | 25,275,189 | CTGGCCAGGAGCCAGTGT | TTAGCCTGAAGGGGAAACTGA |
|  | 2 | 7 | 6 | 25,247,966 | 25,248,338 | TTGACAAACATGGTCCCCTTGA | CAGGACGGGAGGAGCTGGCAGT |
|  | 2 | 8 | 7 | 25,247,479 | 25,247,875 | TTCAGATGGAGCTCCCTCCTGG | AGCAGAACCCACTTCCATCACC |
|  | 2 | 9-10 | 8 | 25,246,539 | 25,247,261 | TGAGACTGACTCTCGAGGCTC | TCCCTAAGCATGGCTTTCCCA |
|  | 2 | 11-12 | 9 | 25,245,931 | 25,246,419 | TGGGAACAAGTTGGAGACCAGG | TCCCATGTCATTCAAACCTTCC |
|  | 2 | 13 | 10 | 25,245,131 | 25,245,462 | ACCCTGGACTCTTTTCTGGCTG | AAGTCTACATCACACGCACACC |
|  | 2 | 14 | 11 | 25,244,399 | 25,244,763 | GGTCATGTCTTCAGGGCTTAGG | TGCTACCTGGAATGGAAAGACC |
|  | 2 | 15-16 | 12 | 25,243,823 | 25,244,438 | TAGCCATGCTCCAGACCCGGTC | CCAGAGTTGCCCACACACCTGG |
|  | 2 | 17 | 13 | 25,241,480 | 25,241,828 | AAGATAGGACTTGGGCCTACAG | AATGAAAGGAGGCAAGGGCTGC |
|  | 2 | 18-19 | 14 | 25,240,221 | 25,240,840 | CTCTTTCGTGTCAAAGGACTTC | AATGCAGATGAGACAGGATG |
|  | 2 | 20 | 15 | 25,239,009 | 25,239,319 | GAGAAGTAAGAGGCTTTGAGGC | AGCAGCTAGTCATTCAGCAGAG |
|  | 2 | 21 | 16 | 25,236,820 | 25,237,112 | ACCCTGTGAACTAGTGGCTGCT | GCATTCTCCACACTAGCTGGA |
|  | 2 | 22 | 17 | 25,235,591 | 25,235,926 | TGTCAGGGTTTGGCGAGTACCT | CTGTTCCCAGGACGTTTGTGGA |
|  | 2 | 23 | 18 | 25,234,079 | 25,234,562 | AGGAGTTGGTGGGTGTGAGTGC | CAGGAGATGATGTCCAACCCTT |
| *TP53* | 17 | 4 | 1 | 7,675,826 | 7,676,436 | CCATGGGACTGACTTTCTGC | CTTTGGGACAGGAGTCAGAGA |
|  | 17 | 5-6 | 2 | 7,674,730 | 7,675,339 | GGAGGTGCTTACGCATGTTT | TTGCACATCTCATGGGGTTA |
|  | 17 | 7 | 3 | 7,674,061 | 7,674,353 | CCTGCTTGCCACAGGTCT | GATGAGAGGTGGATGGGTAGT |
|  | 17 | 8-9 | 4 | 7,673,474 | 7,673,985 | GGCTTCTCCTCCACCTACCT | CGGCATTTTGAGTGTTAGACTG |
| *SETBP1* | 18 | 4 |  | 44,951,652 | 44,952,255 | CTTCACCAGCAGCTATGCAC | CGGTGGGAGATTCTGAACAC |

**Reference Genome: Human GRCh38/hg38**
